# Supplementary material for: Colorimetric RT-LAMP Detection of Multiple SARS-CoV-2 Variants and Lineages of Concern Direct from Nasopharyngeal Swab Samples without RNA Isolation
Source: Viruses. 2023 Sep 12;15(9):1910. doi: 10.3390/v15091910 (PMC10537693; doi:10.3390/v15091910)
Supplement: Supplementary file 1 [file viruses-15-01910-s001.zip › viruses-2543374-supplementary.pdf]

Supplemental material

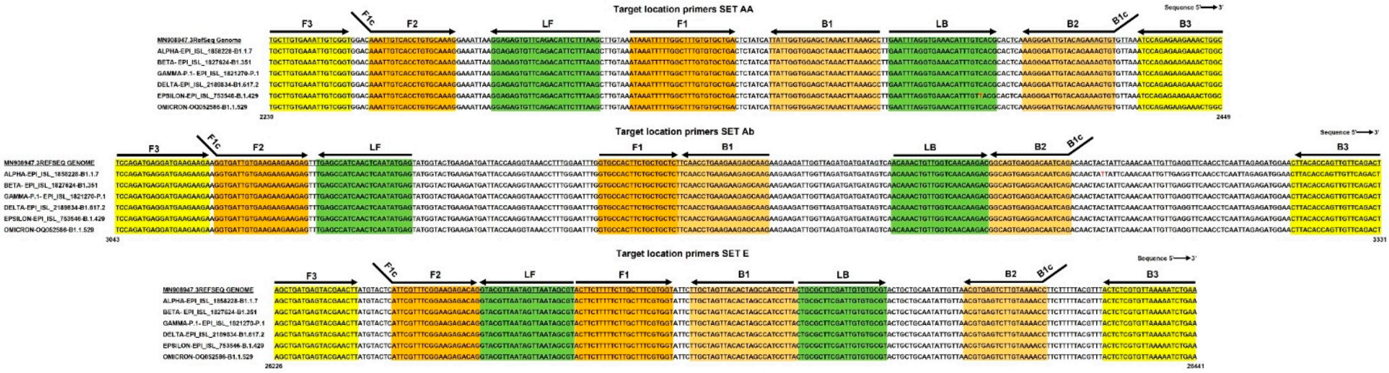

Figure S1. Alignment of LAMP primers with various viral variants

COVID-19 complete genomes	Country	GENBANK ID	% Identity
COVID-19 (Reference Genome)	China	MN908947	
COVID-19		LC522973	100
COVID-19		LC522975	100
COVID-19	China	LR757995	100
COVID-19	China	LR757996	100
COVID-19	China	MN975262	100
COVID-19	USA	MN985325	100
COVID-19	USA	MN988713	100
COVID-19	China	MN996527	100
COVID-19	Australia	MT007544	100
COVID-19	China	MT039873	100
COVID-19	France	MT470114	100
COVID-19	Italy	MT483877	100

Table S1. Percentages of Identity between complete genome Covid-19 (MN908947-Ref) and sequences from different countries from January - May 2020.

High priority pathogens from the same genetic family	GENBANK ID	% Homology Set-E
Human coronavirus 229E	NC_002645	51
Human coronavirus OC43	KX344031	49
Human coronavirus HKU1	KF686346	49
Human coronavirus NL63	JX504050	49
SARS-coronavirus	NC_004718	80
MERS-coronavirus	NC_019843	56

Table S2. Porcentages of Identity between complete genome Covid-19 (MN908947-Ref) and sequences with other coronaviruses

Target region ORF1AA from 2230-2449 bp

ORF 1AA target region from 2230-2449 bp						
	Alpha	Beta	Gamma	Epsilon	Delta	Omicron
	B.1.1.7	B.1.351	P.1	B.1.427, B.1.429	B.1.617.2	B.1.1.529
F3	100 % Homology	100 % Homology	100 % Homology	100 % Homology	100 % Homology	100 % Homology
B3	100 % Homology	100 % Homology	100 % Homology	100 % Homology	100 % Homology	100 % Homology
FIP	100 % Homology	100 % Homology	100 % Homology	100 % Homology	100 % Homology	100 % Homology
BIP	100 % Homology	100 % Homology	100 % Homology	100 % Homology	100 % Homology	100 % Homology
BLP	100 % Homology	100 % Homology	100 % Homology	100 % Homology	100 % Homology	100 % Homology
FLP	100 % Homology	100 % Homology	100 % Homology	100 % Homology	100 % Homology	100 % Homology

Target region ORF1AB from 3043-3331 bp

ORF 1AB target region from 3043-3331 bp						
	Alpha	Beta	Gamma	Epsilon	Delta	Omicron
	B.1.1.7	B.1.351	P.1	B.1.427, B.1.429	B.1.617.2	B.1.1.529
F3	100 % Homology	100 % Homology	100 % Homology	100 % Homology	100 % Homology	100 % Homology
B3	100 % Homology	100 % Homology	100 % Homology	100 % Homology	100 % Homology	100 % Homology
FIP	100 % Homology	100 % Homology	100 % Homology	100 % Homology	100 % Homology	100 % Homology
BIP	100 % Homology	100 % Homology	100 % Homology	100 % Homology	100 % Homology	100 % Homology
BLP	100 % Homology	100 % Homology	100 % Homology	100 % Homology	100 % Homology	100 % Homology
FLP	100 % Homology	100 % Homology	100 % Homology	100 % Homology	100 % Homology	100 % Homology

Target region ORF E from 26226- 26441 bp

ORF 1E target region from 26226 - 26441 bp						
	Alpha	Beta	Gamma	Epsilon	Delta	Omicron
	B.1.1.7	B.1.351	P.1	B.1.427, B.1.429	B.1.617.2	B.1.1.529
F3	100 % Homology	100 % Homology	100 % Homology	100 % Homology	100 % Homology	100 % Homology

B3	100 % Homology	100 % Homology	100 % Homology	100 % Homology	100 % Homology	100 % Homology
FIP	100 % Homology	100 % Homology	100 % Homology	100 % Homology	100 % Homology	100 % Homology
BIP	100 % Homology	100 % Homology	100 % Homology	100 % Homology	100 % Homology	100 % Homology
BLP	100 % Homology	100 % Homology	100 % Homology	100 % Homology	100 % Homology	100 % Homology
FLP	100 % Homology	100 % Homology	100 % Homology	100 % Homology	100 % Homology	100 % Homology

Table S3. In silico reactivity analysis of the different variants analyzed for reactivity with the oligonucleotide primers sequences.

Table S4. Clinical samples from Hospital Muñiz, for clinical validation of direct swab testing without RNA purification.

#	ID samples	Type de sample	RT-PCR	Results (CT)	Results HNB-LAMP
1	578685	NSF	Gene Finder (N/E/RdRp)	20,8/18,6/22,01	Positive
2	578684	NSF	Gene Finder (N/E/RdRp)	26,2/24,2/27,5	Positive
3	578710	NSF	Gene Finder (N/E/RdRp)	23,0/21,0/22,7	Positive
4	578716	NSF	Gene Finder (N/E/RdRp)	32,0/29,5/32,0	Positive
5	580882	NSF	Gene Finder (N/E/RdRp)	27,08/28,65/29,97	Positive
6	580873	NSF	Gene Finder (N/E/RdRp)	16,35/18,28/19,70	Positive
7	580871	NSF	Gene Finder (N/E/RdRp)	20,17/22,89/23,61	Positive
8	580868	NSF	Gene Finder (N/E/RdRp)	22,23/23,57/25,25	Positive
9	**580883**	**NSF**	**Gene Finder (N/E/RdRp)**	**31,97/35,05/33,48**	**Negative**
10	585622	NSF	Gene Finder (N/E/RdRp)	20,6/17,9/20,8	Positive
11	585627	NSF	Gene Finder (N/E/RdRp)	30,3/28,4/31,1	Positive
12	585633	NSF	Gene Finder (N/E/RdRp)	21,8/19,7/21,1	Positive
13	585636	NSF	Gene Finder (N/E/RdRp)	32,7/30,7/33,7	Positive
14	585942	NSF	Gene Finder (N/E/RdRp)	23,9/23,0/25,5	Positive
15	585937	NSF	Gene Finder (N/E/RdRp)	19,4/18,9/20,5	Positive
16	585912	NSF	Gene Finder (N/E/RdRp)	24,6/29,3/29,8	Positive
17	585872	NSF	Gene Finder (N/E/RdRp)	22,84/21,69/24,68	Positive
18	585935	NSF	Gene Finder (N/E/RdRp)	18,83/17,85/20,32	Positive
19	585939	NSF	Gene Finder (N/E/RdRp)	16,13/14,50/16,74	Positive
20	**587107**	**NSF**	**Gene Finder (N/E/RdRp)**	**31,61/30,60/31,26**	**Negative**
21	587165	NSF	Gene Finder (N/E/RdRp)	18,26/16,54/19,36	Positive
22	587161	NSF	Gene Finder (N/E/RdRp)	22,87/21,53/23,98	Positive
23	587160	NSF	Gene Finder (N/E/RdRp)	16,33/14,99/17,56	Positive
24	587158	NSF	Gene Finder (N/E/RdRp)	19,86/19,11/21,57	Positive

25	587151	NSF	Gene Finder (N/E/RdRp)	21,58/20,32/23,45	Positive
26	587144	NSF	Gene Finder (N/E/RdRp)	24,12/22,88/25,60	Positive
27	587155	NSF	Gene Finder (N/E/RdRp)	18,68/17,54/20,27	Positive
28	587159	NSF	Gene Finder (N/E/RdRp)	18,79/17,01/20,25	Positive
29	587134	NSF	Gene Finder (N/E/RdRp)	23,19/21,85/23,99	Positive
30	587042	NSF	Gene Finder (N/E/RdRp)	30,4/28,8/31,4	Positive
31	586885	NSF	Gene Finder (N/E/RdRp)	28,6/27,2/29,9	Positive
32	**586774**	**NSF**	**Gene Finder (N/E/RdRp)**	**31,97/29,82/32**	**Negative**
33	586776	NSF	Gene Finder (N/E/RdRp)	29,87/28,30/30,1	Positive
34	588779	NSF	Gene Finder (N/E/RdRp)	24,40/24,79/24,95	Positive
35	588782	NSF	Gene Finder (N/E/RdRp)	19,96/20,50/20,81	Positive
36	588437	NSF	Gene Finder (N/E/RdRp)	21,5/21,3/21,6	Positive
37	**588811**	**NSF**	**Gene Finder (N/E/RdRp)**	**30,64/30,46/31,81**	**Negative**
38	589094	NSF	Gene Finder (N/E/RdRp)	25,10/25,56/24,84	Positive
39	589103	NSF	Gene Finder (N/E/RdRp)	17,74/18,56/19,1	Positive
40	588537	NSF	Gene Finder (N/E/RdRp)	16,95/15,1/16,5	Positive
41	588539	NSF	Gene Finder (N/E/RdRp)	20,95/19,9/20,0	Positive
42	588799	NSF	Gene Finder (N/E/RdRp)	19,15/19,71/20,03	Positive
43	599800	NSF	Gene Finder (N/E/RdRp)	19,64/19,66/19,89	Positive
44	588808	NSF	Gene Finder (N/E/RdRp)	22,04/22,16/22,16	Positive
45	**588811**	**NSF**	**Gene Finder (N/E/RdRp)**	**30,64/30,46/31,81**	**Negative**
46	588820	NSF	Gene Finder (N/E/RdRp)	17,71/17,75/18,11	Positive
47	588822	NSF	Gene Finder (N/E/RdRp)	25,16/26,31/26,70	Positive
48	588828	NSF	Gene Finder (N/E/RdRp)	20,75/20,28/20,46	Positive
49	588781	NSF	Gene Finder (N/E/RdRp)	26,79/26,89/27,09	Positive
50	588462	NSF	Gene Finder (N/E/RdRp)	19,4/18,1/17,9	Positive
51	588470	NSF	Gene Finder (N/E/RdRp)	17,0/15,7/16,7	Positive
52	588471	NSF	Gene Finder (N/E/RdRp)	17,2/15,8/16,6	Positive
53	588526	NSF	Gene Finder (N/E/RdRp)	24,2/23,4/23,7	Positive
54	**591033**	**NSF**	**Gene Finder (N/E/RdRp)**	**29,26/29,51/30,17**	**Negative**
55	591016	NSF	Gene Finder (N/E/RdRp)	19,89/19,82/20,03	Positive
56	591014	NSF	Gene Finder (N/E/RdRp)	17,64/18,94/17,61	Positive
57	591010	NSF	Gene Finder (N/E/RdRp)	17,42/18,10/18,29	Positive
58	590752	NSF	Gene Finder (N/E/RdRp)	25,1/25,6/25,1	Positive
59	590750	NSF	Gene Finder (N/E/RdRp)	20,2/19,0/19,8	Positive
60	590729	NSF	Gene Finder (N/E/RdRp)	28,9/29,1/29,5	Positive
61	590725	NSF	Gene Finder (N/E/RdRp)	16,2/15,0/15,9	Positive
62	590723	NSF	Gene Finder (N/E/RdRp)	20,9/19,5/20.7	Positive
63	590721	NSF	Gene Finder (N/E/RdRp)	19,0/18,2/18,3	Positive
64	590720	NSF	Gene Finder (N/E/RdRp)	26,1/25,5/25,5	Positive
65	590276	NSF	Gene Finder (N/E/RdRp)	20,20/18,25/21,50	Positive
66	590277	NSF	Gene Finder (N/E/RdRp)	25,79/24/26,05	Positive
67	590266	NSF	Gene Finder (N/E/RdRp)	22,26/21,57/23,25	Positive
68	590262	NSF	Gene Finder (N/E/RdRp)	26,88/25,48/28,59	Positive

69	590257	NSF	Gene Finder (N/E/RdRp)	19,47/18,63/20,69	Positive
70	590259	NSF	Gene Finder (N/E/RdRp)	26,82/25,27/26,60	Positive
71	590223	NSF	Gene Finder (N/E/RdRp)	23,18/20,99/22,62	Positive
72	590224	NSF	Gene Finder (N/E/RdRp)	17,64/15,72/18,89	Positive
73	590226	NSF	Gene Finder (N/E/RdRp)	20,72/19,05/20,90	Positive
74	590233	NSF	Gene Finder (N/E/RdRp)	20,95/19,51/21,92	Positive
75	590523	NSF	Gene Finder (N/E/RdRp)	25,09/26,2/26,5	Positive
76	590537	NSF	Gene Finder (N/E/RdRp)	26,1/25,5/25,5	Positive
77	590749	NSF	Gene Finder (N/E/RdRp)	27,7/27,1/27,8	Positive
78	590675	NSF	Gene Finder (N/E/RdRp)	25,1/23,8/25,1	Positive
79	**591018**	**NSF**	**Gene Finder (N/E/RdRp)**	**31,20/30,84/31,89**	**Negative**
80	**591130**	**NSF**	**Gene Finder (N/E/RdRp)**	**30,2/29,5/32,0**	**Negative**
81	591276	NSF	Gene Finder (N/E/RdRp)	20,85/21,37/21,80	Positive
82	591287	NSF	Gene Finder (N/E/RdRp)	21,88/20,82/20,87	Positive
83	591288	NSF	Gene Finder (N/E/RdRp)	14,90/14,91/14,0	Positive
84	591297	NSF	Gene Finder (N/E/RdRp)	24,68/24,65/24,63	Positive
85	591127	NSF	Gene Finder (N/E/RdRp)	26,3/25,6/26,4	Positive
86	591132	NSF	Gene Finder (N/E/RdRp)	25,8/24,6/26,2	Positive
87	591236	NSF	Gene Finder (N/E/RdRp)	26,8/26,0/27,3	Positive
88	591111	NSF	Gene Finder (N/E/RdRp)	29,1/28,6/30,2	Positive
89	591262	NSF	Gene Finder (N/E/RdRp)	25,2/24,2/25,0	Positive
90	591710	NSF	Gene Finder (N/E/RdRp)	28,96/29,54/30,23	Positive
91	591742	NSF	Gene Finder (N/E/RdRp)	29,37/29,25/30,08	Positive
92	591712	NSF	Gene Finder (N/E/RdRp)	24,49/28,35/28,92	Positive
93	591715	NSF	Gene Finder (N/E/RdRp)	23,67/24,47/23,17	Positive
94	591726	NSF	Gene Finder (N/E/RdRp)	20,59/20,16/20,53	Positive
95	591731	NSF	Gene Finder (N/E/RdRp)	26,16/26,32/26,25	Positive
96	591733	NSF	Gene Finder (N/E/RdRp)	20,22/21,09/21,02	Positive
97	591735	NSF	Gene Finder (N/E/RdRp)	21,52/22,97/24,10	Positive
98	591739	NSF	Gene Finder (N/E/RdRp)	24,72/24,29/24,51	Positive
99	**591743**	**NSF**	**Gene Finder (N/E/RdRp)**	**28,57/28,14/28,59**	**Negative**
100	591102	NSF	Gene Finder (N/E/RdRp)	24,9/24,1/25,0	Positive
101	591105	NSF	Gene Finder (N/E/RdRp)	28,0/28,1/29,8	Positive
102	591294	NSF	Gene Finder (N/E/RdRp)	31,01/30,15/30,34	Positive
103	**591841**	**NSF**	**Gene Finder (N/E/RdRp)**	**32,33/31,61/33,81**	**Negative**
104	591845	NSF	Gene Finder (N/E/RdRp)	22,06/19,79/20,28	Positive
105	591846	NSF	Gene Finder (N/E/RdRp)	18,84/17,78/18,31	Positive
106	591850	NSF	Gene Finder (N/E/RdRp)	19,36/19,60/19,56	Positive
107	591866	NSF	Gene Finder (N/E/RdRp)	28,81/29,49/30,23	Positive
108	591867	NSF	Gene Finder (N/E/RdRp)	21,11/19,81/20,69	Positive
109	591874	NSF	Gene Finder (N/E/RdRp)	24,08/23,21/23,21	Positive
110	591875	NSF	Gene Finder (N/E/RdRp)	24,60/23,83/24,52	Positive
111	591890	NSF	Gene Finder (N/E/RdRp)	22,36/21,99/22,09	Positive
112	591954	NSF	Gene Finder (N/E/RdRp)	28,20/27,65/28,01	Positive

113	591962	NSF	Gene Finder (N/E/RdRp)	21,22/20,70/20,84	Positive
114	591961	NSF	Gene Finder (N/E/RdRp)	23,87/24,98/24,62	Positive
115	591960	NSF	Gene Finder (N/E/RdRp)	21,78/21,55/21,82	Positive
116	591965	NSF	Gene Finder (N/E/RdRp)	28,08/28,51/29,81	Positive
117	591967	NSF	Gene Finder (N/E/RdRp)	28,37/29,04/30,9	Positive
118	591972	NSF	Gene Finder (N/E/RdRp)	26,05/25,92/26,09	Positive
119	**591976**	**NSF**	**Gene Finder (N/E/RdRp)**	**31,09/30,60/31,99**	**Negative**
120	591977	NSF	Gene Finder (N/E/RdRp)	22,78/22,66/22,72	Positive
121	591984	NSF	Gene Finder (N/E/RdRp)	23,07/22,49/22,67	Positive
122	591994	NSF	Gene Finder (N/E/RdRp)	27,14/26,88/27,48	Positive
123	591995	NSF	Gene Finder (N/E/RdRp)	16,41/17,51/16,59	Positive
124	591876	NSF	Gene Finder (N/E/RdRp)	24,77/24,72/24,84	Positive
125	592543	NSF	Gene Finder (N/E/RdRp)	25,9/23,5/32,2	Positive
126	592544	NSF	Gene Finder (N/E/RdRp)	15,9/15,9/18,4	Positive
127	592546	NSF	Gene Finder (N/E/RdRp)	23,2/22,7/22,9	Positive
128	592572	NSF	Gene Finder (N/E/RdRp)	26,8/25,9/26,9	Positive
129	592597	NSF	Gene Finder (N/E/RdRp)	28,5/28,2/28,6	Positive
130	592600	NSF	Gene Finder (N/E/RdRp)	25,5/25,3/25,8	Positive
131	592604	NSF	Gene Finder (N/E/RdRp)	26,8/27,2/27,8	Positive
132	592770	NSF	Gene Finder (N/E/RdRp)	27,5/27,5/28,5	Positive
133	592771	NSF	Gene Finder (N/E/RdRp)	24,7/25,1/25,3	Positive
134	592774	NSF	Gene Finder (N/E/RdRp)	19,8/18,9/19,7	Positive
135	**592788**	**NSF**	**Gene Finder (N/E/RdRp)**	**31,7/30,7/32,3**	**Negative**
136	593145	NSF	Gene Finder (N/E/RdRp)	26,5/26,6/26,9	Positive
137	593148	NSF	Gene Finder (N/E/RdRp)	27,1/28,1/28,1	Positive
138	593278	NSF	Gene Finder (N/E/RdRp)	26,1/25,5/25,8	Positive
139	**593231**	**NSF**	**Gene Finder (N/E/RdRp)**	**31,8/30,8/32,5**	**Negative**

**#**	**ID samples**	**Type of samples**	**RT-PCR**	**Results (CT)**	**Results Neokit Plus**
1	578338	NSF	Gene Finder (N/E/RdRp)	Negative	Negative
2	580876	NSF	Gene Finder (N/E/RdRp)	Negative	Negative
3	585944	NSF	Gene Finder (N/E/RdRp)	Negative	Negative
4	585940	NSF	Gene Finder (N/E/RdRp)	Negative	Negative
5	587166	NSF	Gene Finder (N/E/RdRp)	Negative	Negative
6	587163	NSF	Gene Finder (N/E/RdRp)	Negative	Negative
7	587162	NSF	Gene Finder (N/E/RdRp)	Negative	Negative
8	586771	NSF	Gene Finder (N/E/RdRp)	Negative	Negative
9	588436	NSF	Gene Finder (N/E/RdRp)	Negative	Negative
10	589396	NSF	Gene Finder (N/E/RdRp)	Negative	Negative
11	589397	NSF	Gene Finder (N/E/RdRp)	Negative	Negative
12	589398	NSF	Gene Finder (N/E/RdRp)	Negative	Negative

13	589399	NSF	Gene Finder (N/E/RdRp)	Negative	Negative
14	589400	NSF	Gene Finder (N/E/RdRp)	Negative	Negative
15	591030	NSF	Gene Finder (N/E/RdRp)	Negative	Negative
16	591028	NSF	Gene Finder (N/E/RdRp)	Negative	Negative
17	591027	NSF	Gene Finder (N/E/RdRp)	Negative	Negative
18	591026	NSF	Gene Finder (N/E/RdRp)	Negative	Negative
19	591025	NSF	Gene Finder (N/E/RdRp)	Negative	Negative
20	591024	NSF	Gene Finder (N/E/RdRp)	Negative	Negative
21	591023	NSF	Gene Finder (N/E/RdRp)	Negative	Negative
22	591022	NSF	Gene Finder (N/E/RdRp)	Negative	Negative
23	591021	NSF	Gene Finder (N/E/RdRp)	Negative	Negative
24	591032	NSF	Gene Finder (N/E/RdRp)	Negative	Negative
25	591033	NSF	Gene Finder (N/E/RdRp)	Negative	Negative
26	590718	NSF	Gene Finder (N/E/RdRp)	Negative	Negative
27	590719	NSF	Gene Finder (N/E/RdRp)	Negative	Negative
28	591266	NSF	Gene Finder (N/E/RdRp)	Negative	Negative
29	591267	NSF	Gene Finder (N/E/RdRp)	Negative	Negative
30	591714	NSF	Gene Finder (N/E/RdRp)	Negative	Negative
31	591716	NSF	Gene Finder (N/E/RdRp)	Negative	Negative
32	591824	NSF	Gene Finder (N/E/RdRp)	Negative	Negative
33	591825	NSF	Gene Finder (N/E/RdRp)	Negative	Negative
34	591826	NSF	Gene Finder (N/E/RdRp)	Negative	Negative
35	591842	NSF	Gene Finder (N/E/RdRp)	Negative	Negative
36	591847	NSF	Gene Finder (N/E/RdRp)	Negative	Negative
37	591851	NSF	Gene Finder (N/E/RdRp)	Negative	Negative
38	591855	NSF	Gene Finder (N/E/RdRp)	Negative	Negative
39	591856	NSF	Gene Finder (N/E/RdRp)	Negative	Negative
40	591860	NSF	Gene Finder (N/E/RdRp)	Negative	Negative
41	591864	NSF	Gene Finder (N/E/RdRp)	Negative	Negative
42	592542	NSF	Gene Finder (N/E/RdRp)	Negative	Negative
43	592545	NSF	Gene Finder (N/E/RdRp)	Negative	Negative
44	592566	NSF	Gene Finder (N/E/RdRp)	Negative	Negative
45	592601	NSF	Gene Finder (N/E/RdRp)	Negative	Negative
46	592602	NSF	Gene Finder (N/E/RdRp)	Negative	Negative
47	592603	NSF	Gene Finder (N/E/RdRp)	Negative	Negative
48	592605	NSF	Gene Finder (N/E/RdRp)	Negative	Negative
49	592606	NSF	Gene Finder (N/E/RdRp)	Negative	Negative
50	592607	NSF	Gene Finder (N/E/RdRp)	Negative	Negative
51	592610	NSF	Gene Finder (N/E/RdRp)	Negative	Negative
52	592596	NSF	Gene Finder (N/E/RdRp)	Negative	Negative
53	592598	NSF	Gene Finder (N/E/RdRp)	Negative	Negative
